# Change in Patient Flow in the Epilepsy Care Network Due to Novel Coronavirus Infection: An Opportunity to Strengthen Local Interdisciplinary Epilepsy Care With General Physicians

**DOI:** 10.3389/fneur.2020.591423

**Published:** 2020-11-16

**Authors:** Ayataka Fujimoto, Keishiro Sato, Hideo Enoki

**Affiliations:** ^1^Comprehensive Epilepsy Center, Seirei Hamamatsu General Hospital, Hamamatsu, Japan; ^2^Departments of Neurosurgery, Comprehensive Epilepsy Center, Seirei Hamamatsu General Hospital, Hamamatsu, Japan; ^3^Departments of Neurology, Comprehensive Epilepsy Center, Seirei Hamamatsu General Hospital, Hamamatsu, Japan; ^4^Departments of Pediatric Neurology, Comprehensive Epilepsy Center, Seirei Hamamatsu General Hospital, Hamamatsu, Japan

**Keywords:** patient flow, local interdisciplinary epilepsy network, economic crisis, COVID-19, SARS-CoV-2

## Abstract

**Introduction:** Novel coronavirus disease 2019 (COVID-19) infection caused by severe acute respiratory syndrome coronavirus 2 (SARS-CoV-2) is spreading worldwide. We hypothesized that patient flow in epilepsy care would change as a result of the COVID-19 pandemic. The purpose of this study was to compare the number of patients who visited our epilepsy center before and during the first peak of the pandemic.

**Methods:** We recorded the number of patients with epilepsy referred from general physicians (GPs) to our hospital (GP–H group), the number of patients who visited our hospital on a regular basis (R group), and the number of patients referred from our hospital to GPs (H–GP group) between July 2019 and June 2020.

**Results:** A total of 1,839 epilepsy patients made 4,197 visits to our hospital: 979 males and 860 females (age range, 0–94 years; mean age, 37.6 years; median age, 34 years). There were 433 patients in the GP–H group (247 before the pandemic, 186 during the first peak of the pandemic; *p* = 0.008). In the R group, 1,406 patients made 3,764 visits (1,992 visits before the pandemic, 1,772 during the first peak of the pandemic). In the H–GP group, 135 patients were referred to GPs (47 patients before the pandemic, 88 patients during the first peak of the pandemic; *p* = 0.023).

**Conclusion:** Patient flow in the epilepsy care network changed as a result of the COVID-19 pandemic. These changes might present an opportunity to strengthen local interdisciplinary epilepsy care.

## Introduction

As epilepsy is a chronic disease that requires regular medication and continuous medical oversight, it is important to have insight into the impact of the novel coronavirus disease 2019 (COVID-19) on the epilepsy care network. Currently, COVID-19 infection caused by severe acute respiratory syndrome coronavirus 2 (SARS-CoV-2) is spreading worldwide and places massive strain on health services. Around the beginning of February 2020, an outbreak occurred in Japan onboard the Diamond Princess, a British-registered cruise ship that was carrying 3,711 passengers, of whom 712 were infected by SARS-CoV-2. The number of people infected in foreign countries and entering Japan increased from the beginning of March. Peak infection occurred in mid-April, mainly in urban areas, before temporarily declining in mid-May. According to the World Health Organization COVID-19 Dashboard (https://covid19.who.int/region/wpro/country/jp), the second peak period started at the beginning of July 2020, with the highest peak in August.

Daily broadcasts of medical staff in personal protective equipment made a strong impression on the public, who began to refrain from seeking medical services.

Japan has a universal health insurance system with free access and low cost (1). Before the pandemic, patients tended to visit large hospitals that are well-equipped and have full medical services. The COVID-19 mortality rate at the first peak was lower in Japan than in other countries (2–4), perhaps because the Japanese insurance system covers the majority of medical services and is available to everyone (5). However, because there is free access at low cost, it was common before the pandemic for people to demand unnecessary medical services at large hospitals (6). Thus, the ease of access by any patient to treatment at a tertiary level hospital can discourage patients from visiting their local general physician (GP). As this situation is limited to the Japanese system, most studies of the Japanese medical care system have been published in Japanese, and the Japanese situation is not widely known, although one paper sharply described this situation as a “Tragedy of the Commons” (7).

Since the pandemic, however, people have begun to consider that the risk of SARS-CoV-2 infection is higher at a tertiary hospital than at a local clinic, as the likelihood of social contact is greater at a large institution (8). In addition, negative rumors and fake information have dissuaded people from visiting hospitals (9, 10), which is the opposite of the pre-pandemic situation. As patients with epilepsy have psychological as well as physical stress (11), these patients suffer a greater negative impact compared with patients without epilepsy (12). Therefore, patients with epilepsy should be directed to the most appropriate medical service according to professional advice rather than making a subjective decision themselves based on rumors or fake information.

We hypothesized that patient flow in epilepsy care has changed in response to the COVID-19 pandemic. The purpose of this study is to compare the number of patients who visited our epilepsy center before the pandemic and during the first peak.

## Methods

### Study Design and Ethics Approval

The ethics committee of Seirei Hamamatsu General Hospital, Japan, approved the protocol for this retrospective study, which was performed in accordance with the principles of the Declaration of Helsinki. The subjects of the study were identified in a review of the electronic medical records of patients who visited our epilepsy center between July 2019 and June 2020 at the Comprehensive Epilepsy Center, Seirei Hamamatsu General Hospital.

### Clinical Information

We collected information from patients who had visited our epilepsy center between July 2019 and June 2020 because the first half of this period was pre-pandemic and the second half was just within the first peak of the pandemic period in Japan. Patient age was that recorded at the last visit.

### Primary Outcome Measurement

We recorded the number of patients per month who were classified into each of the following three groups: (1) those referred by their GP for their first visit at our hospital (GP–H group), (2) those who visit our hospital on a regular basis (R group), and (3) those referred by our hospital to their GP (H–GP group). When we refer patients to GPs, their epilepsy information is shared between the hospital epilepsy specialists and the regional epilepsy network of GPs using the Epi Passport booklet (13).

### Secondary Outcome Measurement

#### Type of Epilepsy, Seizure Outcome and Volume of Epilepsy Surgeries Performed

We reviewed the types of epilepsy that were referred to GPs and the outcomes of seizure control in patients in the H–GP group. We classified epilepsy type according to the International League Against Epilepsy (ILAE) 2017 (14) criteria as (1) generalized epilepsy, (2) focal epilepsy, (3) focal and generalized combined epilepsy, and (4) self-limited focal epilepsy. We classified seizure outcome into five levels using a modified version of the ILAE classification system: level 1 (seizure free); level 2 (1–3 seizure days/year); level 3 (4 seizure days/year to 50% reduction); level 4 (<50% reduction in seizures); and level 5 (uncountable due to <1 year of follow up) (13, 15). We recorded the ILAE classification for the most recent outcome at the time when they visited our clinic.

We reviewed the number of epilepsy surgeries performed per month and compared the numbers before the pandemic and during the first peak. We compared the first 6 months (July–December 2019, pre pandemic period) with the second 6 months (January–June 2020, first peak of the pandemic).

#### Second Peak Period

For reference, we also compared number of patients in the H-GP group, R-group, GP-H group, and epilepsy surgeries performed per month in the first 3 months (July–September 2020) of the second peak of the pandemic with those performed in the combined pre pandemic period and first peak of the pandemic.

### Statistical Analysis

We used Student's *t*-test to compare the number of patients and the volume of epilepsy surgeries before the pandemic and during the first peak of the pandemic. A *p* < 0.05 was considered statistically significant. All statistical analyses were conducted using Sigma Plot version 14 (Systat Software, San Jose, CA).

## Results

### Clinical Information

Between July 2019 and June 2020, 1,839 patients made 4,197 visits to our epilepsy center. There were 979 males and 860 females [age range, 0–94 years; mean age, 37.6 years; median age, 34 years; standard deviation (SD), 18.3 years].

### Primary Outcome Measurement

#### GP–H Group

A total of 433 patients in the GP–H group first visited our epilepsy center: 247 patients before the pandemic and 186 patients during the first peak of the pandemic. The age range in this group was 0–94 years (mean age, 38.4 years; median age, 34 years; SD, 18.3 years).

**Figure 1:**
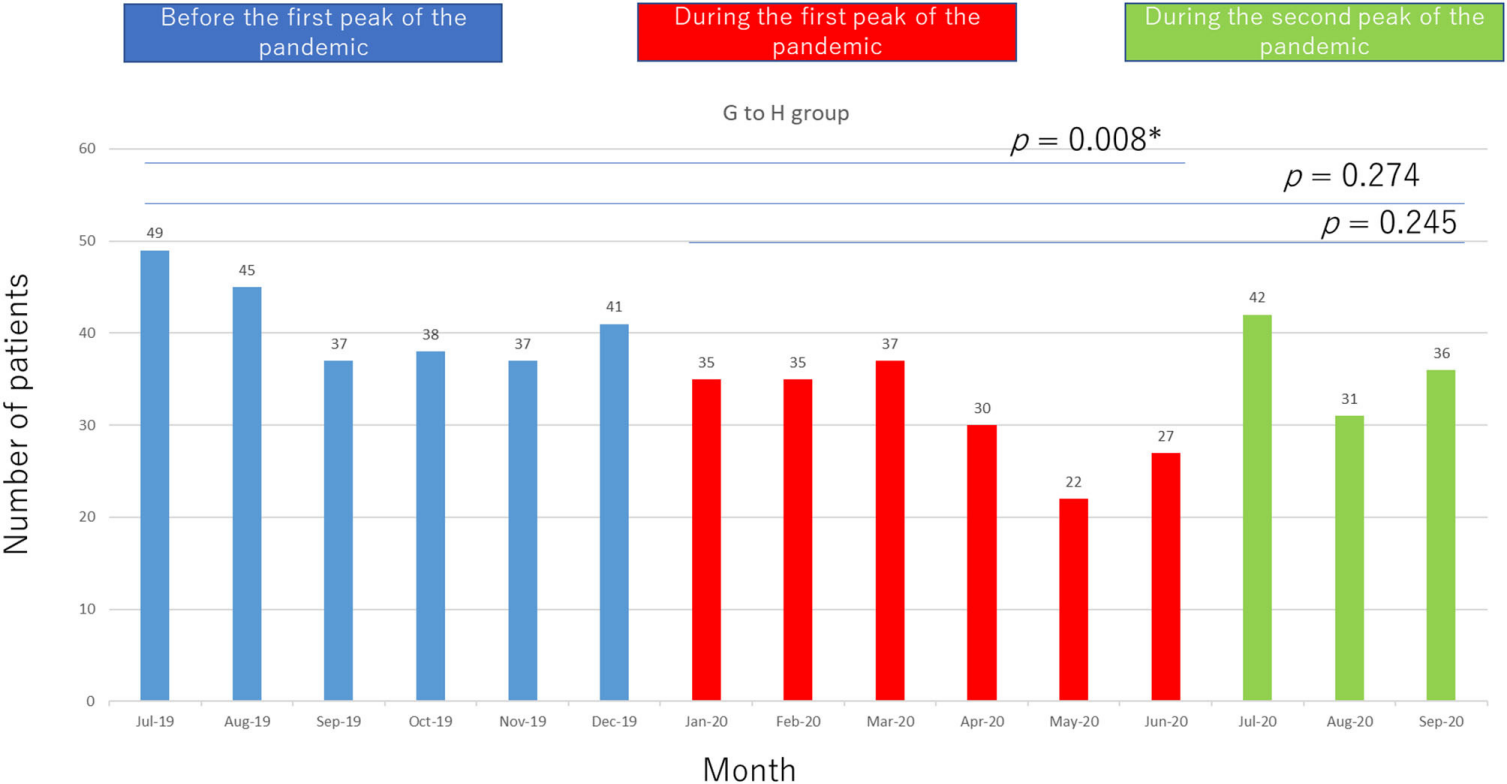
Patient flow from general physicians to our hospital (GP–H group).

The number of patients in this group per month before and during the pandemic is shown in Figure 1 (significant difference, *p* = 0.008).

#### R Group

A total of 1,406 patients in the R group made 3,764 visits to our epilepsy center (age range, 1–87 years; mean age, 37.4 years; median age, 34 years; SD, 18.4 years). There were 1,992 visits before the pandemic and 1,772 visits during the pandemic.

**Figure 2:**
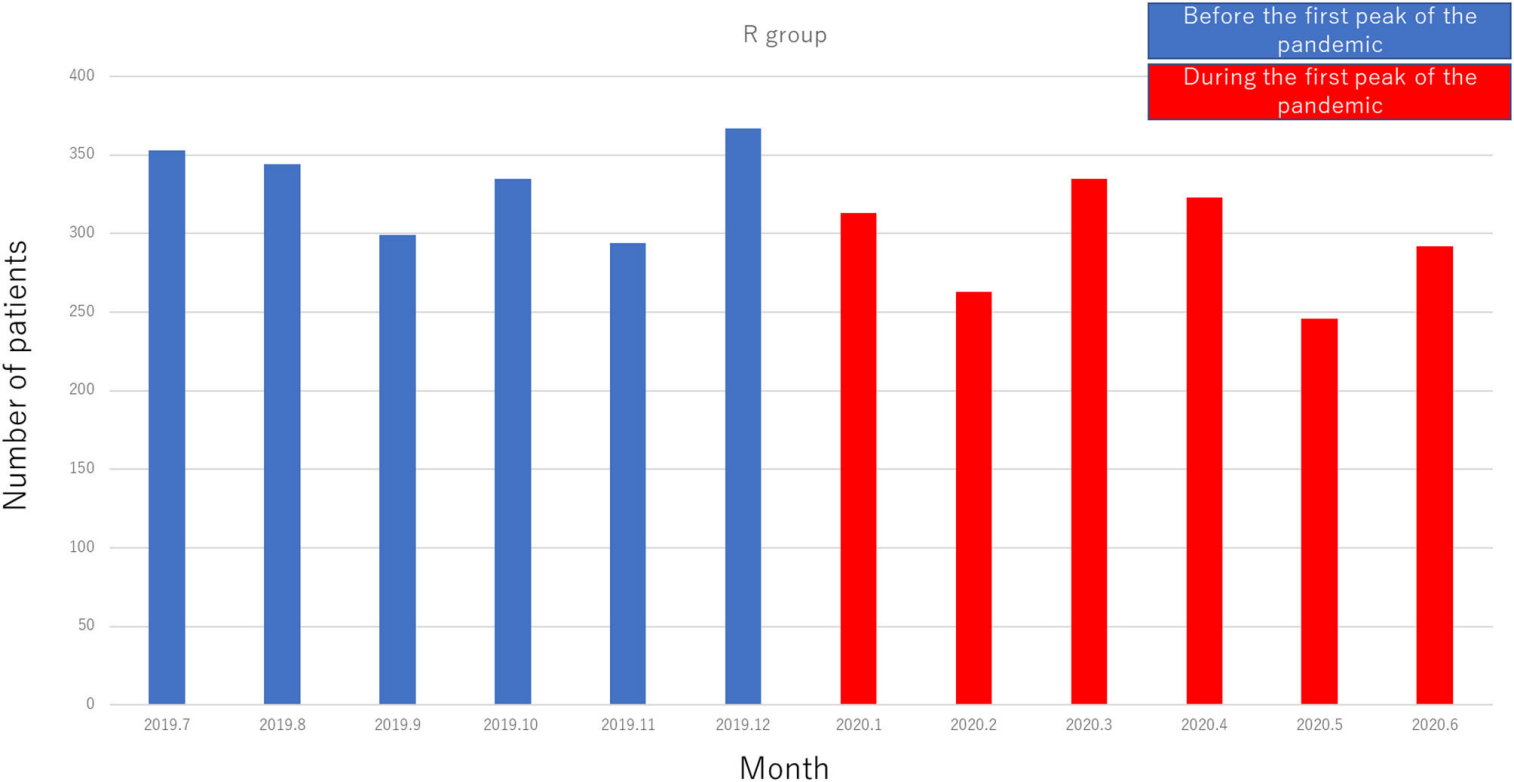
Flow of patients who visit our hospital on a regular basis (R group).

The number of monthly visits before and during the pandemic are shown in Figure 2 (no significant difference).

#### H–GP Group

A total of 135 patients were referred to GPs during the study period. There were 47 patients referred before the pandemic and 88 patients referred during the pandemic. The age range was 0–94 years (mean age, 38.4 years; median age, 34 years; SD, 18.3 years).

**Figure 3:**
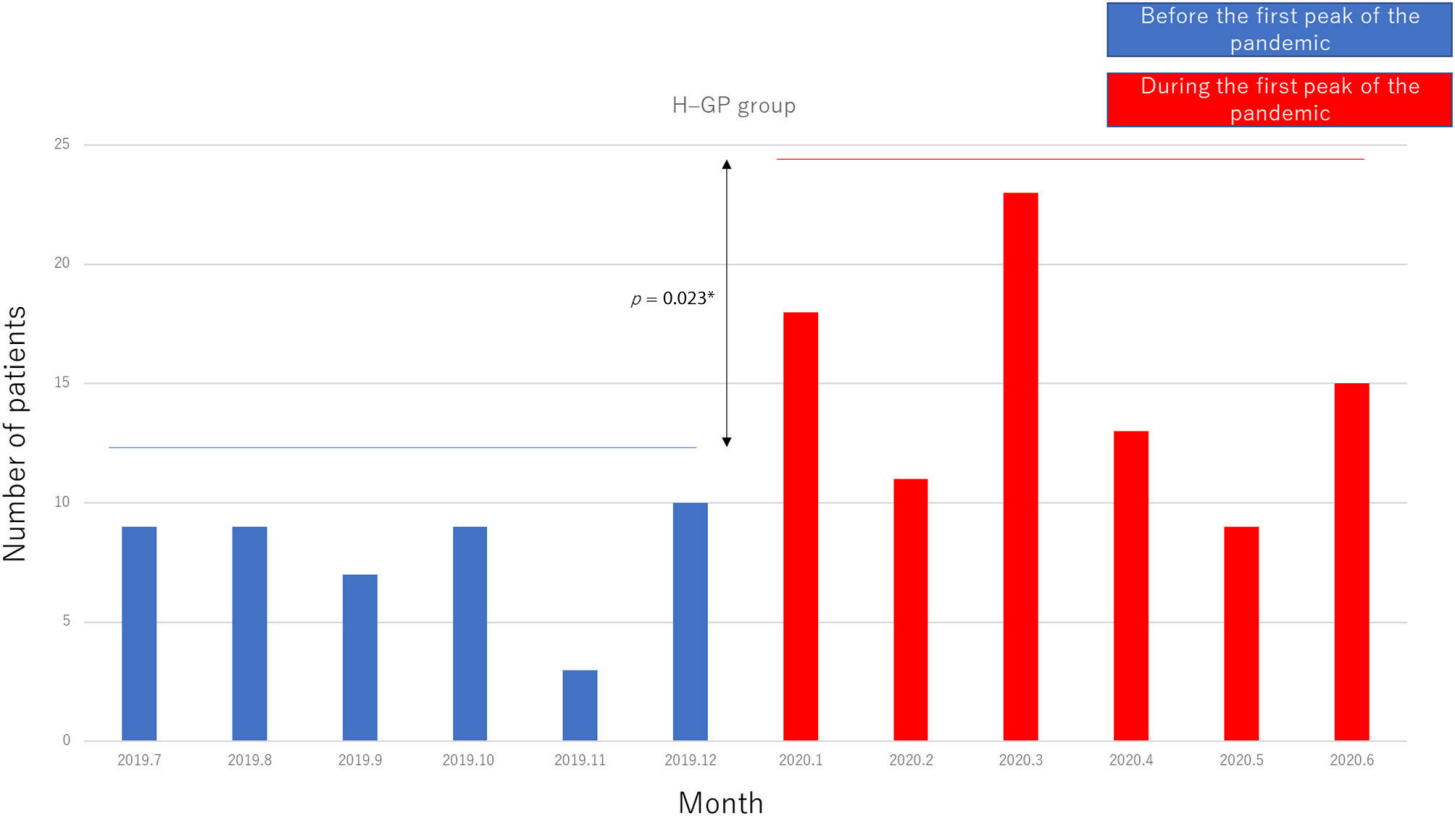
Patient flow from our hospital to general physicians (H–GP group).

The numbers of referrals per month before and during the pandemic are shown in Figure 3 (significant difference, *p* = 0.023).

### Secondary Outcome Measurement

#### Type of Epilepsy, Seizure Outcomes and Volume of Epilepsy Surgeries Performed

**Figure 4:**
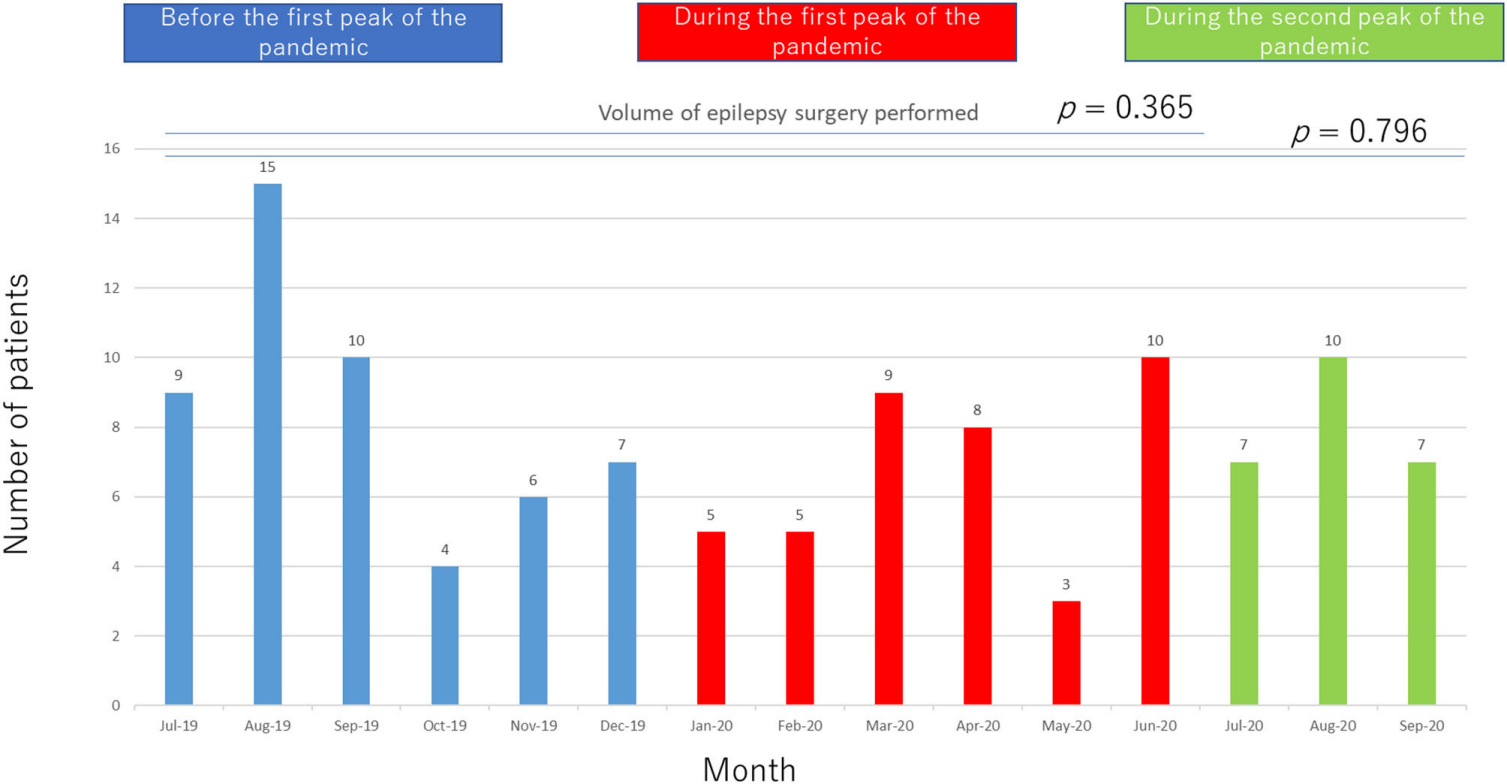
Monthly volumes of epilepsy surgeries performed.

There was no statistically significant difference in the H–GP group before and during the pandemic in terms of type of epilepsy (Table 1). In terms of seizure control outcomes, there was no statistically significant difference in the H–GP group before and during the pandemic (Table 2). We performed 51 epilepsy surgeries before and 40 surgeries during the pandemic (no significant significance) (Figure 4).

**Table 1:** Summary of epilepsy types in the H–GP group before and during the first peak of the pandemic.

	**Before the pandemic**	**During the pandemic**
Generalized epilepsy	14 (30%)	33 (37%)
Focal epilepsy	30 (64%)	52 (58%)
Focal + generalized epilepsy	2 (4%)	4 (5%)
Self-limited focal epilepsy	1 (2%)	0

**Table 2:** Comparison of seizure control outcomes in the H–GP group before and during the first peak of the pandemic.

	**Before the pandemic**	**During the pandemic**
Level 1	42 (89%)	64 (72%)
Level 2	0	3 (3%)
Level 3	1 (2%)	7 (8%)
Level 4	2 (4%)	1 (1%)
Level 5	2 (4%)	14 (16%)

*Level 1 (seizure free); level 2 (1–3 seizure days/year); level 3 (4 seizure days/year to 50% reduction); level 4 (<50% reduction in seizures) and; level 5 (uncountable due to less than a year follow up)*.

#### Second Peak Period

Compared with the data obtained in July 2019–June 2020, data obtained in July–September 2020 showed a greater number of patients in the R group (*p* = 0.017) and a slight increase in the number of patients in the H–GP group during the second peak, but the difference was not statistically significant (*p* = 0.059). In terms of the volume of epilepsy surgeries performed, there was no statistically significant difference among the pre pandemic, first peak, and second peak periods.

## Discussion

The following changes in patient flow to epilepsy care services occurred during the COVID-19 pandemic: (1) the number of patients in the GP–H group decreased, (2) the number of patients in the H–GP group increased, and (3) there was no change in the number of patients in the R group or the volume of surgeries performed for epilepsy during the first peak of the pandemic.

Compared with the first peak, the number of patients in the H–GP group showed a tendency to increase and the number of patients in the R group increased during the second peak of the pandemic.

The number of patients in the GP–H group may have decreased because fewer patients than usual visited a GP during the first peak of the pandemic in Japan, when people were advised not to leave home unless absolutely necessary. It is natural that the number of GP visits would decrease while this policy was in place. We consider that this reduction in patient flow also reduced the number of patients referred to our hospital. People with epilepsy experience social difficulties if they suffer an epileptic seizure in ordinary life; however, the shift to online delivery of many services such as telemetry (11, 16) reduced the need for people to leave their homes. An epileptic seizure is less socially disruptive if it occurs at home than in a public place (e.g., at work, school, while driving or using public transportation, eating out). The arrival of the pandemic heightened the need for streamlining patient flow to epilepsy care services (and also for other diseases); however, economic crisis due to congested patient flow was already imminent in many medical facilities (17–20).

Regional multidisciplinary epilepsy care has been established in our region (Shizuoka, Japan) (13). Although patient flow changed during the pandemic, the number of patients in the R group increased and the volume of epilepsy surgeries performed was maintained even during the second peak, possibly because of the strength of the regional epilepsy network. The increased number of patients in the H–GP group might have contributed to the economic well-being of regional GPs; however, further study is required to prove this.

As seizure outcomes improved over the study period in the H–GP group (Table 2), it is natural that patients would want to reduce the risk of SARS-CoV-2 infection at a large hospital. In addition, a previous study found an increase in the income of epilepsy patients who were seen by local GPs, which was another reason for us to refer patients without seizures to GPs (13). Therefore, it could be said that patients and physicians were thinking similarly at the start of the COVID-19 pandemic. The COVID-19 situation presents an opportunity to streamline patient flow and strengthen local multidisciplinary practice in the epilepsy care network.

There was no significant change in terms of the numbers of patients who visited our department on a regular basis, or in the volumes of epilepsy surgeries. This finding indicates that patient scheduling was efficient and that there was a reduction in irrelevant services provided by our department.

As a limitation of this study, our evaluation was performed during only the first peak of the COVID-19 pandemic, and we cannot anticipate future change in patient flow as the pandemic persists. However, it is known that congested patient flow would have caused an economic crisis in the medical system at some time in the near future. As this is a serious problem facing the medical system, it is important that patient flow is activated appropriately. Whether intensive epilepsy care referral to GPs could activate patient flow and activate the medical economy of the epilepsy network is beyond the scope of the present study, but is worthy of further investigation. There could be some bias in the present study because the periods of observation do not overlap and patient visits during the year are not distributed normally.

## Conclusion

The COVID-19 pandemic has changed patient flow in the epilepsy care network, and this situation presents an opportunity to strengthen multidisciplinary epilepsy care to include local GPs.

## Data Availability Statement

The original contributions presented in the study are included in the article/supplementary materials, further inquiries can be directed to the corresponding author/s.

## Ethics Statement

The studies involving human participants were reviewed and approved by the ethics committee of Seirei Hamamatsu General Hospital, Japan, approved the protocol for this retrospective study, which was performed in accordance with the principles of the Declaration of Helsinki. Written informed consent to participate in this study was provided by the participants' legal guardian/next of kin.

## Author Contributions

We analyzed the data of patients who visited our comprehensive epilepsy center, comprising the departments of Neurosurgery (AF), Neurology (KS), and Pediatric Neurology (HE). All authors contributed equally to data analysis, drafting, and revising the article, are accountable for all aspects of this work, and gave their approval for publication of the final version of the manuscript.

## Conflict of Interest

The authors declare that the research was conducted in the absence of any commercial or financial relationships that could be construed as a potential conflict of interest.
